# Structures and conductivities of stable and metastable Li_5_GaS_4_ solid electrolytes

**DOI:** 10.1039/d1ra03194e

**Published:** 2021-07-21

**Authors:** Takuya Kimura, Chie Hotehama, Atsushi Sakuda, Masahiro Tatsumisago, Akitoshi Hayashi

**Affiliations:** Department of Applied Chemistry, Graduate School of Engineering, Osaka Prefecture University 1-1 Gakuen-cho, Naka-ku Sakai Osaka 599-8531 Japan hayashi@chem.osakafu-u.ac.jp +81-72-2549910 +81-72-2549334

## Abstract

Understanding the differences in the structures and defects in the stable crystalline phase and metastable phase is important for increasing the ionic conductivities of a solid electrolyte. The metastable phase often has higher conductivity than the stable phase. In this study, metastable lithium thiogallate, Li_5_GaS_4_, was synthesized *via* mechanochemistry and stable Li_5_GaS_4_ was obtained by heating the metastable phase. The metastable Li_5_GaS_4_ sample was found to have an antifluorite-type crystal structure with cationic disorder, while the stable phase was found to have a monoclinic crystal structure, similar to that of another solid electrolyte, Li_5_AlS_4_. In both the structures, the Ga^3+^ cations were surrounded by four S^2−^ anions in tetrahedral coordination. The conductivity of the metastable phase was determined to be 2.1 × 10^−5^ S cm^−1^ at 25 °C, which is 1000 times greater than that of the monoclinic phase. The high conductivity of the metastable phase was achieved owing to cation disorder in the crystal structure.

## Abstract 2

A metastable antifluorite-type Li_5_GaS_4_ electrolyte prepared by a mechanochemical process exhibits the highest conductivity of 2.1 × 10^−5^ S cm^−1^ at 25 °C, which is three orders of magnitude higher than that of the heated Li_5_GaS_4_ samples with the stable phase.

## Graphical abstract

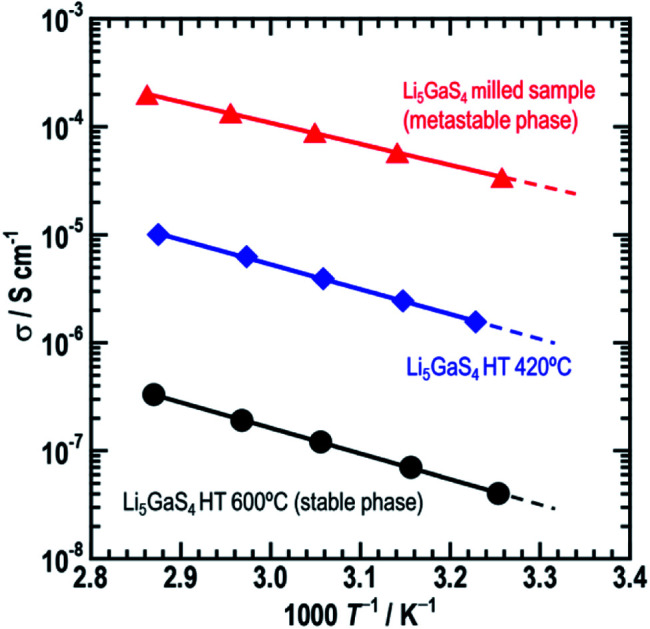

## Introduction

Solid electrolytes, one of the key materials for realizing all-solid-state batteries, are required to have high ionic conductivity, suitable deformability, and high chemical/electrochemical stability. A number of previous studies have suggested that sulfide-based electrolytes meet these requirements. In particular, the ionic conductivities of sulfide electrolytes reach 10^−2^ S cm^−1^ at 25 °C, a value comparable to that of the organic liquid electrolytes used in commercial lithium-ion batteries.^1,2^

Moreover, sulfide electrolytes have better deformability for densification than oxide electrolytes.^3^ So far, various sulfides, such as Li_2_S–P_2_S_5_ glass-based electrolytes,^4–9^ thio-LISICON-type crystals,^10–14^ Li_10_GeP_2_S_12_-type crystals,^1,2,15,16^ and argyrodite-type crystals,^17–20^ have been reported as solid electrolytes. These solid electrolytes have been prepared with a wide range of compositions. For example, the thio-LISICON series have been prepared in the form of Li_4_GeS_4_–Li_3_PS_4_, Li_4_GeS_4_–Li_5_GaS_4_, Li_3_PS_4_–Li_4_SiS_4,_*etc.*^12,14,21^ Further, the crystal structures and conductivities at room temperature of Li_4_GeS_4_, Li_3_PS_4_, and Li_4_SiS_4_, which are the terminal compositions in binary systems, have been reported. As the other terminal composition, Li_5_GaS_4_ has been reported to have a low conductivity of 5.1 × 10^−8^ S cm^−1^ at 100 °C;^14^ however, its crystal structure has not been reported. The Li_5_GaS_4_ crystals are stable at room temperature and can be readily prepared by heating a mixture of the starting materials. On the other hand, glassy and amorphous electrolytes are prepared by melt quenching or a mechanochemical process.^4–8^ In general, glassy and amorphous electrolytes have higher conductivities than the corresponding crystalline phases, because of their higher free volumes. Such glasses are notable precursors for metastable superionic conductive crystals. For example, when the 70Li_2_S·30P_2_S_5_ glass is heated to 240 °C, the superionic conductive phase of Li_7_P_3_S_11_ precipitates as a metastable phase in the amorphous matrix.^6,8,22^ In addition, metastable crystalline phases can be prepared directly *via* mechanochemistry,^23,24^ and metastable crystalline phases often exhibit higher conductivities than the stable crystalline phases. For instance, Li_4_SnS_4_ prepared by heating a mixture of the starting materials has a stable orthorhombic crystal structure,^25,26^ while the Li_4_SnS_4_ sample prepared by the mechanochemical process has the metastable hexagonal crystal structure.^24^ Further, the metastable hexagonal Li_4_SnS_4_ has higher ionic conductivity than the stable orthorhombic Li_4_SnS_4_.

In this study, we focused on the thio-LISICON composition of Li_5_GaS_4_, whose crystal structure has not yet been clarified. In particular, the formation of the metastable phase of Li_5_GaS_4_ by a mechanochemical process (ball milling) was investigated. Subsequently, crystalline phases were obtained by heating the milled metastable Li_5_GaS_4_ sample at different temperatures. The structures of the different crystalline phases were analyzed by X-ray diffraction (XRD) and Raman spectral analyses, and the conductivities of the stable and metastable phases were also examined.

## Experimental section

Li_2_S (Mitsuwa Chemical Co., Ltd., 99.9%) and Ga_2_S_3_ (Kojundo Chemical Lab. Co., Ltd., 99.99%) powders were used as the starting materials for the mechanochemical synthesis of the Li_5_GaS_4_ solid electrolytes. A stoichiometric mixture of 5Li_2_S·1Ga_2_S_3_ (= Li_5_GaS_4_) was mechanochemically processed at 510 rpm for 100 h using a planetary ball mill apparatus (Pulverisette 7; Fritsch GmbH). In this process, 0.5 g of the mixture of the starting materials was milled in a 45 mL zirconia pot with 250 zirconia balls (diameter: 4 mm). After the mechanochemical process, the Li_5_GaS_4_ powder was collected; this sample is hereafter referred to as milled Li_5_GaS_4_. The milled powder was subsequently heated at 420 or 600 °C for 2 h in a dry argon atmosphere; the heat-treated Li_5_GaS_4_ samples are referred to as HT-420 °C or HT-600 °C, respectively. All the steps in the synthesis were carried out in a dry argon atmosphere.

X-ray diffraction (XRD) of the powder was performed on an X-ray diffractometer (SmartLab, Rigaku Corporation) using Cu-Kα radiation. The diffraction patterns were obtained in steps of 0.02° in the 2*θ* range of 10–80° at a scan rate of 10° min^−1^. Rietveld refinement of the XRD patterns was performed using the RIETAN-FP software.^27^ The diffraction data for the Rietveld refinement were collected in steps of 0.02° in the 2*θ* range of 10–130° at a scan rate of 1° min^−1^ using monochromatic Cu-Kα_1_ radiation. For the Rietveld refinement, first, the peak shape, background coefficient, scale factor, and lattice constants were refined. Then, the occupancy was fixed at the stoichiometric composition, and the isotropic displacement parameters of sulfur and gallium were refined. The crystal models were obtained using the VESTA software.^28^

Raman spectroscopic analysis to identify the local structural units in the solid electrolytes was carried out using a Raman spectrophotometer (LabRAM HR-800, HORIBA Ltd.) equipped with a 532 nm diode-pumped solid-state laser.

The ionic conductivity of the solid electrolyte was determined through electrochemical impedance spectroscopy. The impedance data were obtained in the frequency range of 10^7^ to 10^−1^ Hz using an impedance analyzer (SI-1260, Solartron) at an applied AC voltage of 50 mV. The prepared electrolyte powders were pressed at 360 MPa to form pellets at room temperature (∼25 °C). The diameter and thickness of the pellets were approximately 10 mm and 1 mm, respectively. Thin gold films were coated onto the entire surface of the pellets on both the sides to serve as current collectors. The ionic conductivity was measured in the temperature range of approximately 30–75 °C. Activation energies (*E*_a_) were calculated from the slopes of the Arrhenius plots and then the conductivities at 25 °C (*σ*_25°C_) was obtained by extrapolation.

## Results

**Fig. 1:**
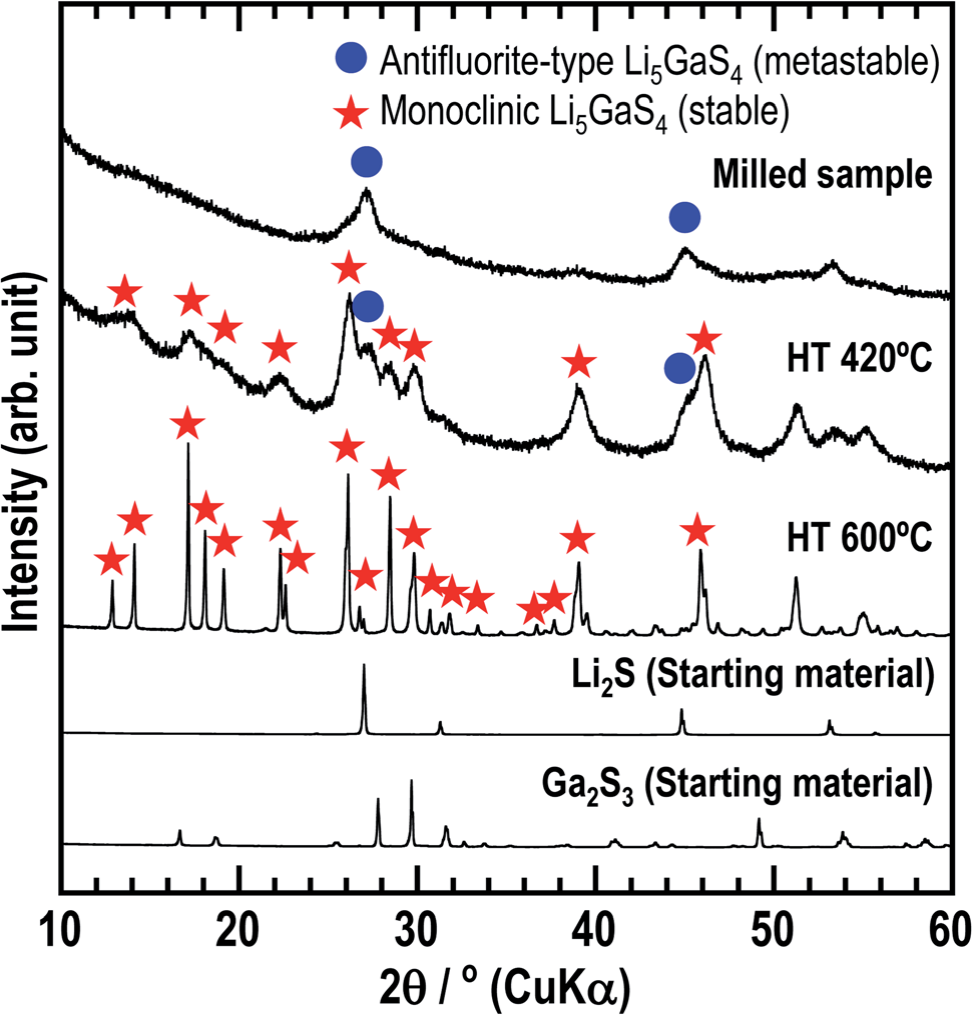
XRD patterns of the three prepared Li_5_GaS_4_ samples and starting materials (Li_2_S and Ga_2_S_3_). The milled sample was prepared by a mechanochemical process, while the HT samples were obtained by heating the milled sample at 420 or 600 °C. Blue circles and red stars indicate the peaks corresponding to the metastable cation-occupancy-disordered antifluorite-type structure and stable monoclinic structure, respectively.

First, the solid electrolyte, milled Li_5_GaS_4_ was prepared by a mechanochemical process. Then, the milled sample was heated at 420 or 600 °C to obtain heated samples (HT-420 °C or HT-600 °C). The milled and heat-treated samples were white powders. Fig. 1 shows the powder XRD patterns of the as-milled and heated Li_5_GaS_4_ samples, along with those of the starting materials. The XRD patterns of the prepared Li_5_GaS_4_ samples contain peaks of unknown phases, as indicated by blue circles or red stars (Fig. 1). The peaks marked by blue circles are similar to those of Li_2_S with an antifluorite-type structure belonging to the cubic system. The XRD pattern of the milled sample only contains the set of peaks marked by blue circles. When the milled sample was heated at 420 °C, two sets of peaks (both the peaks marked by blue circles and by red stars) appeared in the XRD pattern. The peaks marked by red stars could be indexed to the monoclinic structure. Upon heating at a higher temperature of 600 °C, only the peaks marked by red stars were observed. Thus, as the heat-treatment temperature was increased, the peaks indexed to the cubic structure disappeared, while the peaks indexed to the monoclinic structure appeared. These results suggest that the cubic structure is the metastable phase, while the monoclinic structure is the stable phase.

**Fig. 2:**
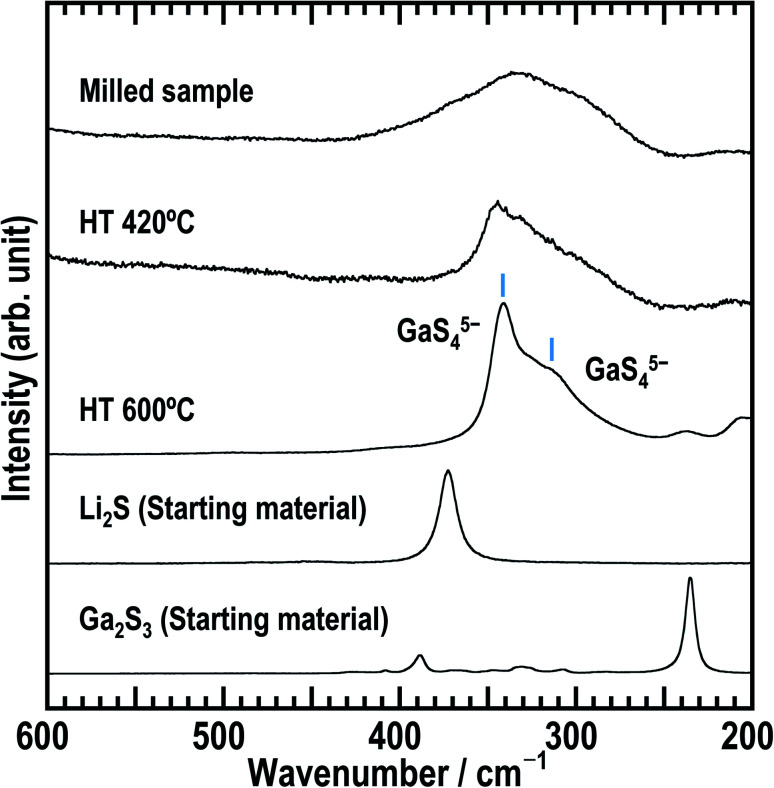
Raman spectra of the three Li_5_GaS_4_ samples and starting materials (Li_2_S and Ga_2_S_3_).

Fig. 2 shows the Raman spectra of as-milled Li_5_GaS_4_ and the heated samples. The spectrum of the milled sample shows a broad peak centered at ∼335 cm^−1^. This peak may contain multiple peaks, but the peak separation is difficult. The peak does not include the component of Ga_2_S_3_ used as the starting material, because its main peak appears at a different wavenumber. Upon heating the sample, the Raman spectrum changed; an asymmetric peak appeared at ∼345 cm^−1^ in the spectrum of HT-420 °C, while two strong peaks appeared at ∼310 and 340 cm^−1^ for HT-600 °C. Note that the bands at ∼310 and 340 cm^−1^ have not been attributed to any units in glasses containing Ga_2_S_3_ in previous studies.^29,30^ Based on the X-ray crystal structure results of HT-600 °C, which will be discussed later, the Raman bands at 310 and 340 cm^−1^ observed for HT-600 °C are assigned to isolated GaS_4_ tetrahedral units.

**Fig. 3:**
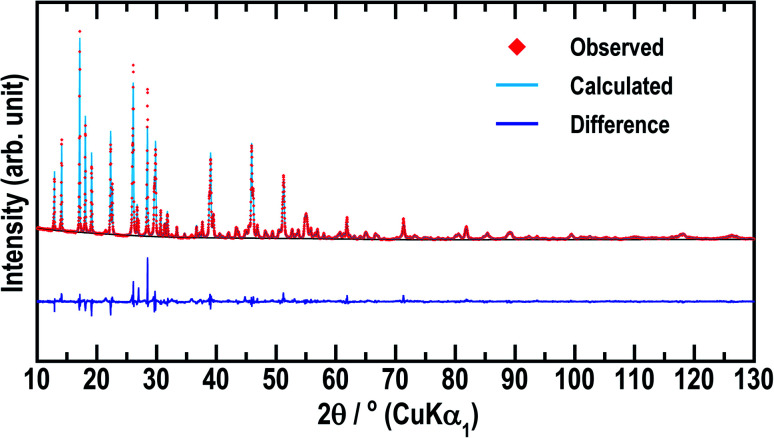
Rietveld refinement of the X-ray diffraction data (Cu-Kα_1_ radiation) for stable monoclinic Li_5_GaS_4_. The red rhombuses, pale blue line, and dark blue line indicate the observed intensity, calculated intensity, and intensity difference, respectively.

**Fig. 4:**
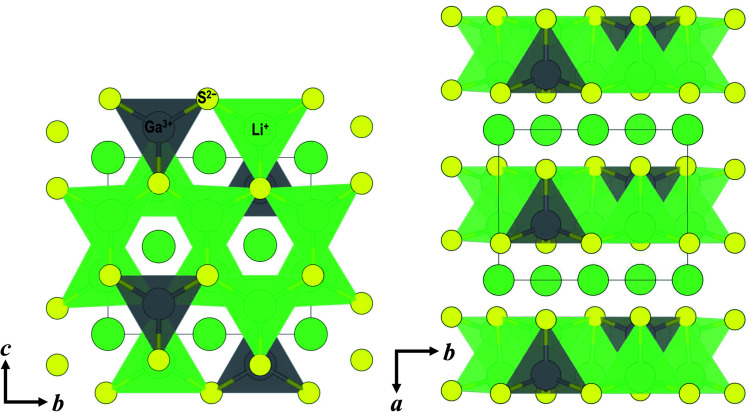
Crystal structures of monoclinic Li_5_GaS_4_ viewed along the *a*-axis (left) and along the *c*-axis (right). Each tetrahedron includes either a lithium or gallium atom in the central position. Lithium atoms in the octahedral interstices are represented by spheres. Green, gray, and yellow spheres represent lithium, gallium, and sulfur, respectively.

To date, only the conductivity of Li_5_GaS_4_ has been reported,^14^ but not its crystal structure. Among the materials with the composition of 5Li_2_S·1M_2_S_3_ (= Li_5_MS_4_; M = B, Al, Ga, In, and Tl), only the crystal structure of Li_5_AlS_4_ has been analyzed in detail.^13^ The similarity of the XRD patterns of Li_5_AlS_4_ and Li_5_GaS_4_ was exploited to identify the crystal structure of Li_5_GaS_4_. The Rietveld refinement results for Li_5_GaS_4_ are presented in Fig. 3 and Table 1, and the crystal structure of Li_5_GaS_4_ is shown in Fig. 4. Li_5_GaS_4_ has the tetrahedral sites of lithium and gallium, and the octahedral site of lithium. In the Rietveld refinement of the XRD pattern of the HT-600 °C sample, the parameters reported for Li_5_AlS_4_ (*P*2_1_/*m* (space group No. 11), *a* = 6.2488 Å, *b* = 7.8369 Å, *c* = 6.8583 Å, *β* = 90.333°) were used as the initial structural parameters, and then the parameters were refined using the Le Bail method. In the Rietveld refinement, the occupancy of all atoms and the atomic displacement parameters of Li were not refined. The full profile and Rietveld fitting of Li_5_GaS_4_ clearly reveal that the refined structure is almost accurate except for the difference in peak intensity at about 28°, which is probably due to the partial occupancy of gallium at the lithium site. Further analysis by neutron diffraction or single crystal X-ray diffraction is required to determine the lithium and gallium occupancy in detail.

Crystallographic data of stable monoclinic Li_5_GaS_4_ obtained by the Rietveld refinement of the X-ray (Cu-Kα_1_ radiation) diffraction. Fractional coordinates and occupancies for Li_5_GaS_4_. *m*W denotes the integrated combination of the multiplicity and Wyckoff letter^a^

**Table:** 

Crystal system	Monoclinic
Space group	*P*2_1_/*m* (no. 11)
Lattice parameter	*a* = 6.262 (1) Å, *b* = 7.857 (2) Å, *c* = 6.852 (2) Å, *α* = *γ* = 90°, *β* = 90.21 (1)°
Volume	*V* = 337.135 (130) Å^3^, *Z* = 2

a *R*_wp_ = 9.370, *R*_F_ = 1.162, *R*_B_ = 3.676, *S* = *R*_wp_/*R*_e_ = 2.4003.

**Table:** 

Site	*m*W	*x*	*y*	*z*	Occ.	*U* Å^−2^
S1	2*e*	0.7539 (5)	1/4	0.8549 (6)	1	0.0120 (15)
S2	2*e*	0.2651 (5)	1/4	0.1749 (6)	1	0.0042 (11)
S3	4*f*	0.7557 (3)	0.0101 (3)	0.3322 (4)	1	0.0096 (9)
Ga	2*e*	0.6326 (3)	1/4	0.1706 (3)	1	0.0087 (7)
Li1	4*f*	0.3515 (28)	0.5167 (16)	0.3290 (27)	1	0.0127
Li2	2*e*	0.3524 (45)	1/4	0.8413 (44)	1	0.0127
Li3	2*e*	0.0008 (40)	1/4	0.4967 (36)	1	0.0127
Li4	2*a*	0	0	0	1	0.0127

**Fig. 5:**
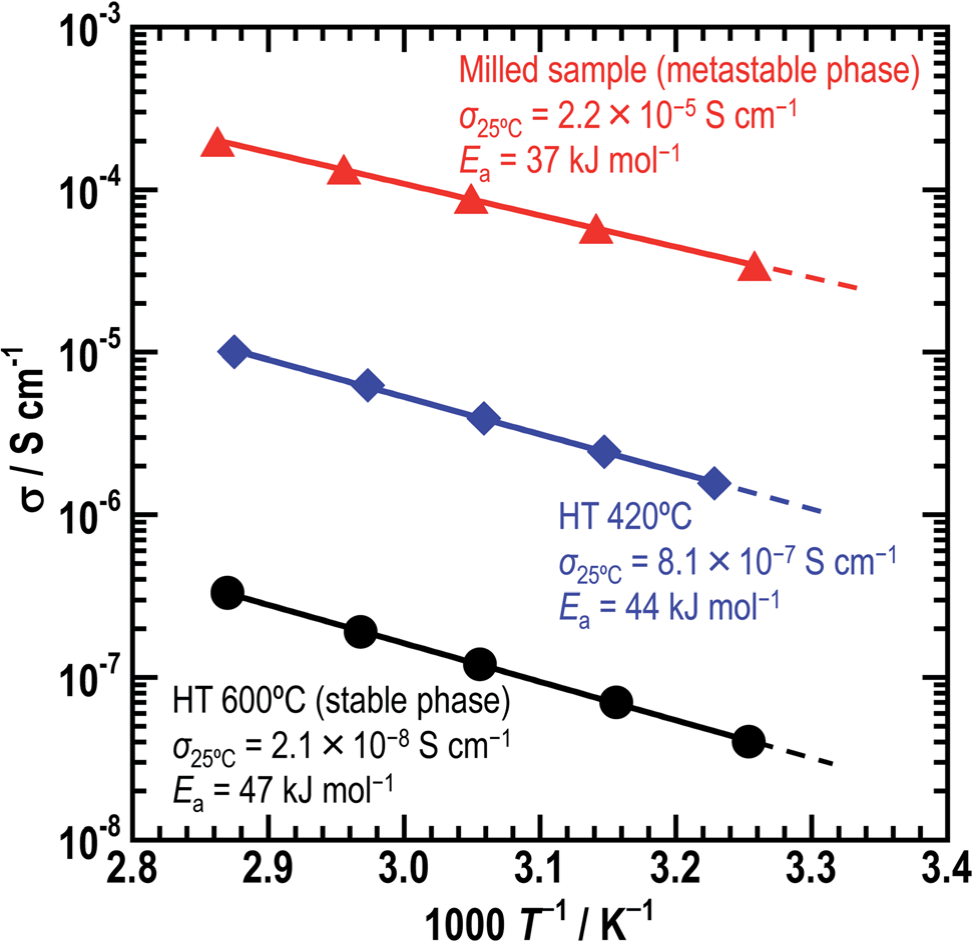
Temperature-dependence of the conductivities of the three Li_5_GaS_4_ samples. Red triangles, blue rhombuses, and black circles indicate the conductivities of the milled, HT-420 °C, and HT-600 °C samples, respectively. The activation energies were calculated using the Arrhenius equation, *σ* = *A* exp(−*E*_a_/*RT*), where *A*, *σ*, *E*_a_, *R*, and *T* are the pre-exponential factor, conductivity, activation energy, gas constant, and measurement temperature, respectively.

Fig. 5 shows the temperature-dependence of the conductivities of the three Li_5_GaS_4_ samples. At the composition of Li_5_GaS_4_, the conductivities differed according to the heating temperature. The milled sample showed the highest conductivity of 2.2 × 10^−5^ S cm^−1^ at 25 °C among the three prepared samples. The ionic conductivities of HT-420 °C and HT-600 °C at 25 °C were 8.1 × 10^−7^ and 2.1 × 10^−8^ S cm^−1^, respectively. The activation energies of the milled sample, HT-420 °C, and HT-600 °C were calculated to be 37, 44, and 47 kJ mol^−1^, respectively.

## Discussion

In the XRD patterns of the prepared Li_5_GaS_4_ samples (Fig. 1), the peaks of the milled sample are comparable to those of Li_2_S, one of the two starting material. However, the Raman band of crystalline Li_2_S at ∼370 cm^−1^ was not clearly observed in the Raman spectrum of the milled Li_5_GaS_4_ sample. Thus, the peaks marked by blue circles in the XRD patterns were assigned to the antifluorite-type crystal structure, and not to the starting material Li_2_S. Although a detailed analysis of the metastable crystal phase is difficult because of the broad XRD peaks, the observed XRD peaks can be attributed to a new metastable phase with the antifluorite-type crystal structure, which has eight tetrahedral sites for the cation surrounded by four anions in a unit cell. In general, in a cation-disordered crystal structure, the cation sites are randomly occupied by cations or defects. In the case of antifluorite-type Li_5_GaS_4_, lithium cations, gallium cations, and defects randomly occupy the eight cation sites. The metastable antifluorite-type Li_5_GaS_4_ has a similar structure to the monoclinic Li_5_GaS_4_ (Fig. 4) because both phases are composed of isolated GaS_4_ tetrahedra. The ionic radii of lithium and gallium cations in the antifluorite-type crystal are however significantly different; the sizes of Li^+^ and Ga^3+^ are 0.59 Å and 0.47 Å under tetrahedral coordination (*n* = 4; *n* is the coordination number), respectively.^31^ The cation sites of the antifluorite-type structure seem to have a high tolerance to the size of the cations. Such a cation-disordered phase has been previously reported for Li_4_SnS_4_ and Li_2_TiS_3_.^24,32^ In the crystal structure of hexagonal Li_4_SnS_4_, the tetrahedral sites are occupied by Li^+^ (*r*_ionic_ (*n* = 4): 0.59 Å) and Sn^4+^ (*r*_ionic_ (*n* = 4): 0.55 Å). In the mechanochemically synthesized Li_2_TiS_3_, the octahedral sites are occupied by cations of different sizes, *viz.*, Li^+^ (*r*_ionic_ (*n* = 6): 0.76 Å) and Ti^4+^ (*r*_ionic_ (*n* = 6): 0.605 Å). These results indicate that cation disorder is possible not only in a structure with cations of similar sizes but also in structures containing cations of different sizes prepared by the mechanochemical process. Thus, the mechanochemical process is effective in the preparation of disordered structures, and the obtained disordered structures are metastable and have faster ionic conduction than the thermodynamically stable phases. The milled Li_5_GaS_4_ sample with a metastable crystal structure has higher conductivity (2.2 × 10^−5^ S cm^−1^) than the heated samples with a stable crystal structure (2.1 × 10^−8^ S cm^−1^) at 25 °C, as shown in Fig. 5. The XRD peaks attributable to the metastable phase in the milled Li_5_GaS_4_ sample are broad, and they are mainly due to small crystallite size and/or disordered structure of the metastable phase. The sample possibly includes amorphous phase, which may contribute to the high conductivity of the milled Li_5_GaS_4_ sample.

In the structural analysis of the stable Li_5_GaS_4_ crystal, the structural parameters were refined using the parameters of Li_5_AlS_4_. The lattice volumes of Li_5_GaS_4_ and Li_5_AlS_4_ are 337.135 and 335.8537 Å^3^, respectively. Although the difference between the cation radii of Ga^3+^ (0.47 Å) and Al^3+^ (0.39 Å)^31^ for tetrahedral coordination is large, the difference in their volumes is small. This assumption is reasonable considering the packed structure of the anions and cations. If we consider the ions as rigid spheres for simplicity, the critical ionic radius ratio for tetrahedral coordination (*r*_cation_/*r*_anion_) is 0.225. In the MS_4_ tetrahedral unit, the critical cation radius is 0.41 Å, when the anion radius of S^2−^ is 1.84 Å.^31^ The critical cation radius is larger than the radius of Al^3+^, and the tetrahedral structure of AlS_4_ is unstable, according to simple numerical calculations. However, in fact, the tetrahedral unit is formed because of the distortion of the tetrahedral symmetry and the distortion of the electron clouds of the sulfide anions. The Ga–S and Al–S distances are 2.31 and 2.28 Å in the same tetrahedral unit, respectively. The volume of GaS_4_ tetrahedra is 6.34 Å^3^, larger than that of AlS_4_ tetrahedra (6.04 Å^3^). The crystal structure of Li_5_GaS_4_ is illustrated in Fig. 4. The filling structure of the polyhedra (GaS_4_, LiS_4_, and LiS_6_) in Li_5_GaS_4_ is the same as that in Li_5_AlS_4_. The crystal of Li_5_GaS_4_ consists of two layers, the MS_4_ (M = Li, Ga) layer and LiS_6_ layer, which are stacked alternately. All the tetrahedral interstices are occupied by Li or Ga in the MS_4_ layer, while all the octahedral interstices are occupied by Li in the LiS_6_ layer. In these crystals with completely filled sites, the ionic conductivity is usually low.

In crystalline ionic conductors, the site vacancy and number of carriers are important for fast ionic conduction. Considering the ionic conduction in the crystal structure, monoclinic Li_5_GaS_4_ simultaneously has the advantage of high lithium content and the disadvantage of fully occupied sites. The conductivity of Li_5_GaS_4_ HT-600 °C is 2.1 × 10^−8^ S cm^−1^ at 25 °C. Note that Li_5_GaS_4_ has been previously reported to have a low conductivity of 5 × 10^−8^ S cm^−1^ at 100 °C.^14^ In comparison, the conductivity of HT-600 °C (monoclinic) at 100 °C, as estimated using the Arrhenius equation, is ∼20 times higher at 9.8 × 10^−7^ S cm^−1^.^14^ Compared to those of other stoichiometric thio-LISICON materials, the conductivity of monoclinic Li_5_GaS_4_ is lower; for instance, it is lower than those of γ-Li_3_PS_4_ (3 × 10^−7^ S cm^−1^),^10^ Li_4_SiS_4_ (5 × 10^−8^ S cm^−1^),^11^ Li_4_GeS_4_ (3 × 10^−7^ S cm^−1^),^14^ and Li_4_SnS_4_ (7 × 10^−5^ S cm^−1^),^25^ but higher than those of Li_5_AlS_4_ (9.7 × 10^−9^ S cm^−1^ at 50 °C)^13^ and Li_3_SbS_4_ (4.8 × 10^−9^ S cm^−1^).^33^ The differences in the conductivities is due to the lithium content, vacancy of lithium sites, and central cation–sulfide anion interaction. Understanding the differences in the conductivities of thio-LISICON is challenging because of various factors, such as the differences in their ductility and relative density of pellets. The HT-420 °C sample, which has a mixed structure consisting of the antifluorite-type and monoclinic crystals, shows a conductivity of 8.1 × 10^−7^ S cm^−1^, which is higher than that of HT-600 °C. This results from the precipitation of the metastable phase. Thus, among the three samples prepared in this study, the as-milled sample with the metastable antifluorite-type crystal phase has the highest conductivity, while the HT-600 °C sample with the stable monoclinic crystal phase has the lowest conductivity. The results clearly indicate that the antifluorite-type crystal is more suitable for ionic conduction than the monoclinic crystal.

## Conclusions

In this study, sulfide antifluorite-type structure is proposed as a new flamework for ionic conduction. A metastable Li_5_GaS_4_ solid electrolyte was prepared by a mechanochemical process and subsequently transformed into a stable Li_5_GaS_4_ solid electrolyte by heat treatment. The mechanochemically processed sample had the metastable antifluorite-type phase. When heated at 600 °C, the phase transformed to the stable monoclinic one, similar to that of Li_5_AlS_4_. The conductivity of the milled sample (metastable antifluorite-type phase) was determined to be 2.1 × 10^−5^ S cm^−1^ at 25 °C, which is three orders of magnitude higher than that of the heated sample with the stable phase. Thus, it is concluded that the metastable phase is a more suitable structure for ionic conduction than the stable phase at the composition of Li_5_GaS_4_. The results of this study extend research toward understanding sulfide electrolytes with cation-disordered metastable phases and cation-ordered stable phases, and contribute to the development of solid electrolytes with high ionic conductivity.

## Author contributions

T. K., A. S., and A. H. designed the experiments and wrote the paper. T. K. synthesized and characterized the electrolytes. T. K. and C. H. performed the crystal analysis. A. S., T. M., and A. H. supervised the study. All of the authors discussed the results and commented on the manuscript.

## Conflicts of interest

There are no conflicts to declare.

## Supplementary Material
